# Comparison of a preservative-free nonsteroidal anti-inflammatory drug and preservative-free corticosteroid after uneventful cataract surgery: multicenter, randomized, evaluator-blinded clinical trial

**DOI:** 10.1097/j.jcrs.0000000000000841

**Published:** 2021-10-08

**Authors:** Seonjoo Kim, Byung-Yi Ko, Jae Woong Koh, Eun Chul Kim, Hong Kyun Kim, Young Joo Shin, Jong-Suk Song, Do Hyung Lee, Ji Eun Lee, Hyung Keun Lee, So-Hyang Chung, Hyun Seung Kim

**Affiliations:** From the Department of Ophthalmology, Seoul St. Mary's Hospital, College of Medicine, The Catholic University of Korea, Seoul, South Korea (S.J. Kim, Chung, H.S. Kim); Department of Ophthalmology, Konyang University College of Medicine, Daejeon, South Korea (Ko); Department of Ophthalmology, Chosun University College of Medicine, Gwangju, South Korea (Koh); Department of Ophthalmology, Bucheon St. Mary’s Hospital, College of Medicine, The Catholic University of Korea, Bucheon, South Korea (E.C. Kim); Department of Ophthalmology, School of Medicine, Kyungpook National University, Daegu, South Korea (H.K. Kim); Department of Ophthalmology, Kangnam Sacred Heart Hospital, Hallym University College of Medicine, Seoul, South Korea (Shin); Department of Ophthalmology, Korea University College of Medicine, Seoul, South Korea (Song); Department of Ophthalmology, Ilsan Paik Hospital, Inje University College of Medicine, Goyang, South Korea (D.H. Lee); Department of Ophthalmology, Pusan National University Yangsan Hospital, Pusan National University School of Medicine, Yangsan, South Korea (J.E. Lee); Department of Ophthalmology, Gangnam Severance Hospital, Yonsei University College of Medicine, Seoul, South Korea (H.K. Lee).

## Abstract

To compare the efficacy of nonsteroidal anti-inflammatory drugs (NSAIDs) and steroidal eyedrops for inflammation management after cataract surgery using slitlamp indicators.

**Setting:**

11 eye centers in South Korea.

**Design:**

Randomized prospective multicenter study with a blinded evaluator.

**Method:**

In 125 (250 eyes) patients who underwent cataract surgery, bromfenac sodium hydrate 0.1% (NSAID group) was applied twice a day in 1 eye, whereas the other eye was treated with fluorometholone 0.1% (steroid group), 4 times a day for 4 weeks postoperatively. The primary efficacy outcome was the presence of anterior chamber cells and flare at 1 week postoperatively. Anterior chamber cells and flare at 4 to 8 weeks, corrected distance visual acuity, central corneal thickness, conjunctival hyperemia, dry eye parameters, foveal thickness, and ocular and visual discomfort were evaluated as secondary outcomes.

**Results:**

At week 1, residual anterior chamber inflammation was not statistically significantly different between the groups (−1.03 ± 1.27 vs −0.95 ± 1.24, *P* = .4850). However, the NSAID group recovered from conjunctival hyperemia more rapidly than the steroid group (0.30 ± 0.52 vs 0.44 ± 0.81, *P* = .0144 at week 1). The increase in central corneal thickness in the NSAID group was less than that in the steroid group 1 week postoperatively (7.87 ± 22.46 vs 29.47 ± 46.60 μm, *P* < .0001). The change in foveal thickness in the NSAID group was significantly less than that in the steroid group (18.11 ± 68.19 vs 22.25 ± 42.37 μm, *P* = .0002). Lower levels of postoperative ocular and visual discomfort were reported in the NSAID group than in the steroid group under treatment.

**Conclusions:**

Preservative-free bromfenac was as effective as preservative-free fluorometholone eyedrops in anterior chamber inflammation control and showed better signs and symptoms after cataract surgery.

## Abstract 2

Preservative-free bromfenac was as effective as preservative-free fluorometholone eyedrops in anterior chamber inflammation control and showed better signs and symptoms profile after cataract surgery.

Cataract is a major cause of blindness, in addition to glaucoma and macular degeneration. The prevalence of cataracts has rapidly increased in the aging population. Currently, surgery is the only treatment of cataracts, and cataract surgery is one of the most commonly performed surgeries in the ophthalmic field. Developments in cataract surgery, such as phacoemulsification using a minimal incision and advancements in surgical equipment and intraocular lenses (IOLs) and ophthalmic viscosurgical devices, have reduced the risk for physical trauma and complications associated with the surgery. However, postoperative inflammation, which can be caused by changes in the ocular surface and tear film due to intraoperative manipulations or preservatives (eg, benzalkonium chloride) in eyedrops, can result in discomfort and delayed recovery.^1^ In addition, poorly controlled inflammation can cause complications such as endophthalmitis, cystoid macular edema (CME), and posterior capsule opacity. Furthermore, proper management is important because complications may prevent the restoration of normal vision.^2^

Steroids are well-known anti-inflammatory agents that act through mechanisms such as inhibition of inflammatory response mediator production.^3^ Steroids have been effectively used for inflammatory eye diseases, including management of inflammation after cataract surgery. However, steroid use is associated with a high risk for side effects, such as increased intraocular pressure (IOP), and increased risk for infection.^4^ Clinically, prednisolone, dexamethasone, fluorometholone, and loteprednol are the commonly used steroids. Among them, fluorometholone is the most widely used because of its relatively low risk for side effects such as increased IOP.

Nonsteroidal anti-inflammatory drug (NSAIDs) are another class of anti-inflammatory agents. Unlike steroid eyedrops, NSAIDs do not increase the IOP or the risk for infection. Moreover, NSAIDs induce mitotic inhibition and have analgesic effects during surgery,^5^ and the benefits of preventing CME by NSAIDs after cataract surgery have been confirmed.^6,7^ Currently, ketorolac, diclofenac, and bromfenac are used clinically. Bromfenac increases the permeability of ocular tissues and extends the duration of action of amfenac owing to the addition of a bromide group to the existing formulation of amfenac.^8^ It reaches the retina in concentrations that are effective for the prevention of CME and can be applied only twice per day, whereas other similar preparations must be applied 3 or 4 times.^9^ A single-use, preservative-free version of bromfenac eyedrops has been recently released, which may avoid the risks for ocular surface damage and unstable tear film caused by preservatives.

Several studies have evaluated the efficacy and safety of NSAIDs compared with those of steroids for the management of inflammation after cataract surgery.^6,7,10^ A 2017 Cochran meta-analysis reported that NSAIDs have a superior anti-inflammatory effect on anterior flare as measured by flow cytometry, which is almost impossible to use clinically.^11,12^ However, no conclusion could be drawn based on the number of anterior cells as a clinical index. Although the results regarding corneal edema were insufficient, NSAIDs are more effective than steroids for preventing CME.^11^

Therefore, this study aimed to compare the effects of NSAIDs with those of steroids for the management of inflammation after cataract surgery based on anterior inflammatory and corneal conjunctival indicators using a slitlamp examination, a method that can be applied in a real healthcare environment.

## METHODS

### Study Population and Procedure

In this multicenter (11 centers) blinded prospective study, patients older than 50 years with age-related cataracts who planned to undergo cataract surgery in both eyes were enrolled. The cataracts were of grades 3 to 4 with nuclear sclerosis, and the preoperative endothelial cell count of the patients was ≥1500 cells/mm^2^. This study was conducted in accordance with the ethical principles of the Declaration of Helsinki and Good Clinical Practice Guidelines and was approved by the Institutional Review Board of each participating center, including Yeouido St. Mary's Hospital (Institutional Review Board No. SC18MCDV009), before initiation of the study. In addition, this trial was registered at the Current Research Information System (http://cris.nih.go.kr) and the World Health Organization International Clinical Trials Registry Platform (www.who.int/ictrp). The trial registration number is KCT0003340.

Patients with a history of eye trauma or corneal transplantation were excluded. The other exclusion criteria included congenital cataract, aniridia, iris atrophy, pseudoexfoliation syndrome, and any retinal disease that can affect vision, such as diabetic retinopathy, epiretinal membrane, and macular edema. Patients were also excluded if they were or should have been administered other eyedrops for therapeutic purposes within 2 weeks of cataract surgery; had chronic or recurrent ocular inflammatory disease (such as uveitis or scleritis); or had glaucoma, allergy, or dry eye disease requiring treatment other than artificial tears. In addition, patients with systemic diseases that might affect the results (eg, uncontrolled diabetes or hypertension) and who had received systemic NSAIDs or steroids within 2 weeks of cataract surgery or those who would need them during the clinical trial period were excluded. Patients were also excluded if they experienced surgical complications such as posterior capsular rupture or vitreous prolapse during surgery or if the investigator determined that an active inflammatory treatment was necessary postoperatively. Patients were also excluded at the investigators' discretion for other reasons that may affect the interpretation of the results.

After the initial screening visit, 125 patients met the eligibility criteria. All patients underwent cataract surgery in both eyes within 1 week by the same surgeon and visited the clinic the day after surgery for evaluation of surgical outcomes for each eye. For each patient, the anti-inflammatory drug instilled in each eye (right or left eye) was determined randomly by a sequence generated by an independent statistical service (Seoul CRO, Co., Ltd.) using SAS 9.4 (SAS Institute Inc.) and by permuted stratified block randomization with participating centers as the strata. A dedicated unblinded investigator dispensed the assigned eyedrop for each eye with deliberate instillation instructions and used a subject diary to record the actual daily usage for each eye. Thus, each patient received eyedrops of 1 type of treatment in 1 eye and control eyedrops in the other eye.

In the control eye (steroid group), Fluvin ophthalmic solution 0.1% (single unit dose) (fluorometholone 1.0 mg/mL, Taejoon Pharm. Co., Ltd.) was instilled at a dosage of 1 drop 4 times daily for 4 weeks, and in the treatment eye (NSAID group), Bronuck ophthalmic solution 0.1% (single unit dose, bromfenac sodium hydrate 1.0 mg/mL, Taejoon Pharm. Co., Ltd.) was administered at a dosage of 1 drop twice daily for 4 weeks. In addition, Culevox eyedrops 0.5% (levofloxacin 5.0 mg/mL, Taejoon Pharm. Co., Ltd.) were administered at a dosage of 1 drop 3 times per day from 3 days prior to surgery to 1 month postoperatively in both eyes. If necessary, NewHyalUni 0.15%eyedrops (single use, sodium hyaluronate 1.5 mg/mL, Taejoon Pharm. Co., Ltd.), used as artificial tears, were allowed. The 2 drugs differed in the number of daily eyedrops; therefore, the evaluator was blinded because the blinding of the subjects was limited. However, to maintain objectivity on the patients' questionnaire-based evaluation forms and minimize the possibility of evaluator blindfolding, masking was maintained by packaging the treatment and control drugs in indistinguishable boxes.

### Efficacy and Safety End points

The primary efficacy end point was standardization of uveitis nomenclature (SUN) inflammation grade in the anterior chamber 1 week after treatment with Bronuck or Fluvin. In addition, the SUN inflammation grade, sum of anterior chamber cells, and flare at the end of treatment (4 weeks) and observation (8 weeks) were evaluated, and dichotomous analysis of inflammation resolution defined as anterior inflammation grade zero was performed. The secondary end points included corrected distance visual acuity (logMAR), corneal edema on the Efron grading scale (0 to 4), change in central corneal thickness, bulbar redness on the IER grading scale (0 to 4), corneal conjunctival staining score (Oxford staining score, 0 to 5), and tear breakup time (TBUT) at weeks 1, 4, and 8. In addition, changes in foveal thickness from preoperatively to the end of treatment (4 weeks) and any posterior capsular opacification at the end of observation (8 weeks) were evaluated. To assess patient satisfaction with surgery, ocular discomfort (0 to 9; sum of superficial pain, foreign body or gritty sensation, or other unspecified discomfort) and visual discomfort (0 to 9; sum of visual disturbance, glare, or halo) were surveyed by evaluating disturbances in daily life (0 to 3) for each eye. Daily pain experiences in each eye were recorded in the subject diary. At each visit during the treatment period, drug instillation tolerance was evaluated using a 10-point visual analog scale, followed by a description of the most annoying symptom.

Considering the indications of this study and the use of a steroid eyedrop as the active control, the dropout rates for active inflammation management and IOP were evaluated as safety end points along with adverse events (AEs) during the treatment period.

### Statistical Analysis

Power analysis was performed to determine the sample size. All statistical analyses were performed using SAS 9.4 64-bit (SAS Inc.) software.

Descriptive statistics (mean, standard deviation, median, minimum, and maximum values) are presented for the continuous variables at each timepoint in the NSAID and steroid groups. A paired *t* test or Wilcoxon signed-rank test, if necessary, was performed to assess intragroup changes preoperatively and postoperatively and for intergroup comparisons, considering the correlation between eyes reported in a previous study.^13^ To evaluate categorical efficacy, the frequency and ratio are presented. The chi-square test or Fisher exact test, if necessary, or McNemar test was performed for intragroup and intergroup comparisons.

For the primary end point, noninferiority of the treatment eyedrop was assessed by calculating the upper limit of the 97.5% 1-sided CI for the intergroup difference with a noninferiority margin of 0.62.

The full analysis set (FAS) was defined as all randomized patients with the primary efficacy data, and the per-protocol set (PPS) included all eligible patients without major protocol deviations. The PPS was the primary population for all efficacy analyses. The FAS was used for confirmatory purposes. The safety set comprised all patients who, according to their patient diaries, received the study treatment at least once.

## RESULTS

### Patients

**Figure 1.**
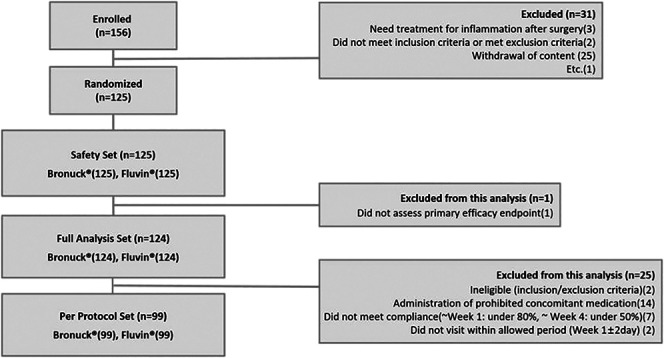
Patient disposition.

Among the 156 enrolled patients, 125 were randomized after confirmation of the eligibility of both eyes; these 125 patients received medications in each eye to determine drug safety. The FAS included 124 patients who received at least 1 dose of study medication and provided data for the primary efficacy end point. From the FAS, 99 patients who completed the trial without major protocol deviations were included in the PPS analysis (Figure 1). The mean age of the patients was 70.10 ± 8.45 years. Thirty-nine patients (39.39%) were men. All IOLs were acrylate IOLs; among the 99 IOLs, 87 were monofocal and 12 were multifocal IOLs. The same type of IOL was implanted in both eyes of the same person. There were no statistically significant differences in visual acuity, IOP, corneal conjunctiva, and macular and cataract severity preoperatively and phacoemulsification status during surgery between both groups (Table 1).

**Table 1.** Demographic and Baseline Clinical Characteristics of Patients in the Per-Protocol Set.

Demographic	NSAID group (n = 99) mean ± SD (range)	Steroid group (n = 99) mean ± SD (range)	*P* value^a^
UDVA (logMAR)	0.42 ± 0.26 (0.00, 1.00)	0.44 ± 0.23 (0.00, 1.00)	.5750
CDVA (logMAR)	0.36 ± 0.29 (0.00, 1.00)	0.40 ± 0.30 (0.00, 1.00)	.3173
IOP (mm Hg)	14.24 ± 3.35 (7.00, 30.00)	14.31 ± 3.99 (6.00, 38.00)	.8750
Keratoconjunctiva			
Conjunctival injection	0.38 ± 0.78 (0.00, 3.00)	0.37 ± 0.75 (0.00, 3.00)	1.0000
Oxford staining	0.25 ± 0.58 (0.00, 3.00)	0.22 ± 0.51 (0.00, 3.00)	.4531
TBUT(s)	6.27 ± 2.10 (1.50, 13.00)	6.23 ± 2.08 (2.00, 13.00)	.8421
CCT (μm)	539.47 ± 40.51 (322.00, 625.00)	534.65 ± 46.72 (237.00, 624.00)	.0902
ECD (cells/mm^2^)	2641.81 ± 369.27 (1768.00, 3921.00)	2623.32 ± 378.61 (1512.00, 3937.00)	.6099
Macula			
Foveal thickness (μm)	245.50 ± 39.58 (21.00, 322.00)	245.14 ± 40.14 (80.00, 344.00)	.5621
Lens			
Nucleus (N)	3.32 ± 0.47 (3.00, 4.00)	3.31 ± 0.47 (3.00, 4.00)	1.0000
Cortex (C)	3.07 ± 1.29 (0.00, 5.00)	3.11 ± 1.31 (0.00, 5.00)	.5444
Posterior capsule (P)	0.00 ± 0.00 (0.00, 0.00)	0.00 ± 0.00 (0.00, 0.00)	
Phacoemulsification			
Time (s)	32.79 ± 25.94 (0.00, 90.00)	33.73 ± 24.81 (1.00, 90.00)	.3438
Power (%)	22.02 ± 15.36 (0.00, 63.00)	21.81 ± 15.35 (0.00, 55.00)	.2303

CCT = central corneal thickness; ECD = endothelial cell density; TBUT = tear breakup time;

a Wilcoxon signed-rank test

### Efficacy Results

The change in SUN inflammation grade of the anterior chamber was −1.03 ± 1.27 in the NSAID group and −0.95 ± 1.24 in the steroid group from postoperative day 1 to week 1, representing a significant decrease in both eyes (*P* < .0001) (Table 2). The percentage of patients in whom inflammation disappeared in the anterior chamber (inflammation grade: 0) was 69.70% (69 eyes) in the NSAID group and 70.71% (70 eyes) in the steroid group at 1 week and 93.94% (93 eyes) in the NSAID group and 91.92% (91 eyes) in the steroid group at 4 weeks (Table 3). At week 8, all patients in both groups, except 1 patient, had an inflammation grade of 0.

**Table 2.** Change in Inflammation Grade of Anterior Chamber in the Per-Protocol Set.

Period	NSAID group (n = 99) mean ± SD (range)	Intragroup *P* value^a^	Steroid group (n = 99) mean ± SD (range)	Intragroup *P* value^a^	Intergroup *P* value^a^
1 d	1.24 ± 1.29 (0.00, 6.00)		1.25 ± 1.34 (0.00, 6.00)		.9889
1 wk	0.22 ± 0.40 (0.00, 2.00)		0.30 ± 0.65 (0.00, 4.00)		.1744
4 wk	0.04 ± 0.17 (0.00, 1.00)		0.06 ± 0.25 (0.00, 2.00)		.7031
8 wk	0.01 ± 0.05 (0.00, 0.50)		0.01 ± 0.10 (0.00, 1.00)		1.0000
Δ 1 wk to 1 d	−1.03 ± 1.27	<.0001	−0.95 ± 1.24	<.0001	.4850
Δ 4 wk to 1 d	−1.20 ± 1.31	<.0001	−1.19 ± 1.30	<.0001	.7503
Δ 8 wk to 1 d	−1.24 ± 1.30	<.0001	−1.24 ± 1.32	<.0001	.9476

a Wilcoxon signed-rank test

**Table 3.** Patients With Anterior Inflammation Grade 0 in the Per-Protocol Set.

Period	NSAID group (n = 99) n (%)	Steroid group (n = 99) n (%)	Intergroup *P* value^a^
1 d	10 (10.10)	11 (11.11)	.5637
1 wk	69 (69.70)	70 (70.71)	.7815
4 wk	93 (93.94)	91 (91.92)	.4795
8 wk	98 (98.99)	98 (98.99)	1.0000

a Intergroup (paired 2-sample): McNemar test

The increase in central corneal thickness 4 weeks postoperatively in the NSAID group was also significantly less than that in the steroid group (5.32 ± 18.93 vs 10.16 ± 23.28 μm, *P* = .0071) (Table 4). Furthermore, the NSAID group had lower conjunctival hyperemia at 1 week postoperatively than that preoperatively (Δ week 1 to preoperatively: −0.08 ± 0.53), whereas the steroid group had higher conjunctival hyperemia at 1 week postoperatively than that preoperatively (Δ week 1 to preoperative: 0.07 ± 0.64). Four weeks postoperatively, both groups showed statistically significant improvements compared with preoperatively (*P* value at 4 weeks, NSAID group = .0078, steroid group = .0086; at 8 weeks, NSAID group = .0009, steroid group = .0005) (Table 5). The change in foveal thickness at the end of anti-inflammatory treatment (4 weeks) in the NSAID group was 18.11 ± 68.19 μm, which was significantly lesser than that in the steroid group (22.25 ± 42.37 μm, *P* < .0002) (Supplemental Table 1, http://links.lww.com/JRS/A491).

**Table 4.** Change in Central Corneal Thickness in the Per-Protocol Set.

Period	NSAID group (n = 99) mean ± SD (range)	Intragroup *P* value	Steroid group (n = 99) mean ± SD (range)	Intragroup *P* value	Intergroup *P* value
Preop	539.47 ± 40.51 (322.00, 625.00)		534.65 ± 46.72 (237.00, 624.00)		.0902^b^
1 wk	547.34 ± 48.81 (258.00, 667.00)		564.12 ± 48.41 (465.00, 701.00)		<.0001^b^
4 wk	547.01 ± 37.49 (473.00, 644.00)		547.85 ± 39.85 (455.00, 637.00)		.2419^b^
8 wk	544.20 ± 37.00 (455.00, 630.00)		543.39 ± 39.73 (433.00, 626.00)		.9627^b^
Δ 1 wk to preop	7.87 ± 22.46	.0007^a^	29.47 ± 46.60	<.0001^b^	<.0001^b^
Δ 4 wk to preop	5.32 ± 18.93	.0065^a^	10.16 ± 23.28	<.0001^a^	.0071^a^
Δ 8 wk to preop	4.73 ± 31.75	.1677^b^	8.75 ± 40.95	.0017^b^	.0811^b^

preop = preoperative

Mean ± SD (minimum, maximum)

a Paired *t*-test

b Wilcoxon signed-rank test

**Table 5.** Change in Conjunctival Injection in the Per-Protocol Set.

	NSAID group (n = 99) mean ± SD (range)	Intragroup *P* value^a^	Steroid group (n = 99) mean ± SD (range)	Intragroup *P* value^a^	Intergroup *P* value^a^
Preop	0.38 ± 0.78 (0.00, 3.00)		0.37 ± 0.75 (0.00, 3.00)		1.0000
1 d	0.86 ± 1.01 (0.00, 4.00)		0.87 ± 0.99 (0.00, 4.00)		.8959
1 wk	0.30 ± 0.52 (0.00, 2.00)		0.44 ± 0.81 (0.00, 3.00)		.0144
4 wk	0.24 ± 0.57 (0.00, 3.00)		0.24 ± 0.61 (0.00, 3.00)		1.0000
8 wk	0.20 ± 0.53 (0.00, 3.00)		0.18 ± 0.46 (0.00, 2.00)		.6250
Δ 1 wk to preop	−0.08 ± 0.53	0.1316	0.07 ± 0.64	0.3202	.0110
Δ 4 wk to preop	−0.14 ± 0.50	0.0078	−0.13 ± 0.51	0.0086	1.0000
Δ 8 wk to preop	−0.18 ± 0.54	0.0009	−0.19 ± 0.53	0.0005	1.0000

preop = preoperative

a Wilcoxon signed-rank test

There was no statistically significant difference between the groups regarding corneal Oxford staining, TBUT, corrected distance visual acuity, and the incidence of posterior capsule turbidity.

Subjective ocular and visual acuity discomfort after cataract surgery showed a tendency to increase significantly more in the steroid group than that in the NSAID group at week 1; however, there was no statistically significant change at the end of the observation period (Supplemental Table 2, http://links.lww.com/JRS/A492). Based on the daily subject diary record, the incidence of subjective pain was significantly less in the NSAID group compared with that in the steroid group (26.26% [26 eyes] vs 35.35% [35 eyes]. *P* = .0290), and there was no statistically significant difference between the groups regarding the time required to resolution of subjective pain. Drug compliance during the administration period was higher in the NSAID group than in the steroid group (96.93 ± 11.23% vs 96.30 ± 11.34%; *P* < .0001).

### Safety Results

The safety analysis set comprised 125 patients who received at least 1 dose of the investigational product. No patients were withdrawn because of the need for active inflammation management after the administration of clinical trial drugs. In addition, there was a statistically significant decrease in postoperative IOP in both groups compared with preoperative IOP, and no clinically significant increase in IOP or difference between both eyes was observed.

Regarding eye-related AEs in the safety set, 6 cases (4.80%) were investigated in 6 patients, none of which were serious. Adverse drug reactions were observed in both eyes of the same patient with dry eye in 1 eye (0.80%) (Supplemental Table 3, http://links.lww.com/JRS/A493). Therefore, there was no statistically significant difference in the incidence of eye-related AEs or adverse drug reactions between both eyes (all *P* = 1.0000), and only one case (0.80%) of CME was identified in the steroid group.

## DISCUSSION

This was a multicenter study designed to compare the effects of NSAIDs and corticosteroids after cataract surgery based on anterior inflammatory indicators using slitlamps, which can easily be applied in a real healthcare environment. Bronuck, bromfenac sodium hydrate solution 0.1% that was administered twice a day, was associated with a similar level of anterior chamber inflammation to that with Fluvin, fluorometholone solution 0.1% administered 4 times a day. In addition, Bronuck was shown to improve various corneal conjunctival indicators and discomfort after cataract surgery.

Accordingly, studies have been conducted to address whether a corticosteroid used for the management of inflammation after cataract surgery can be replaced with an NSAID,^6,7,10^ and meta-analysis results and the ESCRS PREMED study report 1 have been published.^11,12,14^ In a 2017 Cochrane meta-analysis, less flare of the anterior chamber and a lower risk for developing CME were observed in the group that received an NSAID alone compared with the group that received a corticosteroid alone. However, it was unclear whether the number of cells was higher or lower and whether there was a higher incidence of corneal swelling. Moreover, in the studies comparing a combination of NSAID plus corticosteroid with corticosteroid alone, a lower risk for developing CME was observed in the group that received combination treatment. However, few studies have described intraocular cell and flare and corneal edema. In this study, the grade of anterior chamber inflammation was evaluated based on the SUN criteria under slitlamps. In the ESCRS PREMED study report 1, the proportion of patients with residual anterior chamber inflammation confirmed by slitlamp examination was 4.0% in the bromfenac group and 4.4% in the dexamethasone group at 6 weeks postoperatively.^14^ Similarly, in our study, we confirmed that the effects of bromfenac 0.1% and fluorometholone 0.1% on anterior chamber inflammation were equivalent.

In addition, our study showed an increase in central corneal thickness in the NSAID group, which was significantly less than the increase in the steroid group at 1 week and 4 weeks postoperatively (*P* < .0001).

Regarding dry eye signs and symptoms after cataract surgery, bromfenac showed noninferior results compared with fluorometholone because of its anti-inflammatory action. Fujishima et al. have shown that bromfenac sodium ophthalmic solution improved the dryness of the eye and signs of dry eye disease, especially, TBUT and corneal surface punctate erosion, through its anti-inflammatory effects.^15^

The limitations of this study include the possibility that a patient older than 50 years would not be able to properly administer corticosteroids or NSAIDs. In this regard, 1 researcher, not an efficacy evaluator, was designated to explain to each patient the number of eyedrops for each eye according to a randomization result. Patients were provided a subject diary, in which instructions for use were written and in which the patients recorded the number of eyedrops administered to each eye on a daily basis. In addition, at each visit, it was confirmed whether the eye was properly secured by assessing the number of eyedrops recorded for each eye. As a result, there were a maximum of 5 suspected patients who reversed the number of eyedrops based on the randomization. The results of the PPS analysis excluding these 5 patients were similar to those of FAS analysis; therefore, the effect on the results was considered limited.

Since this study included only patients who underwent simple cataract surgery, the results cannot be applied to patients at a high risk for inflammation postoperatively. Additional research is needed to investigate the benefits of NSAIDs regarding corneal edema, conjunctival hyperemia, and improved eye discomfort postoperatively compared with those of other strong corticosteroids such as dexamethasone, because fluorometholone, which was administered as control eyedrops, is a weak steroid. In addition, except for patients with dry eye disease who required treatment, there were no significantly abnormal signs of keratoconjunctival staining compared with those preoperatively; however, the fact that the eye discomfort index and TBUT increased after 4 weeks of anti-inflammatory treatment should be referred to in the future studies.

Considering that bromfenac 0.1% (Bronuck) has an equivalent level of anti-inflammatory activity to fluorometholone 0.1% (Fluvin) but a better effect on improving various keratoconjunctival signs and symptoms after cataract surgery and better compliance and tolerability, it can be used effectively for the treatment of inflammation after simple cataract surgery.

**WHAT WAS KNOWN**

- Steroids are well-known anti-inflammatory agents that act through mechanisms such as inhibition of inflammatory response mediator production; however, steroid use is associated with a high risk for side effects, such as increased IOP and increased risk for infection.
- NSAIDs are more effective than corticosteroids in preventing cystoid macular edema and corneal edema.

**WHAT THIS PAPER ADDS**

- The effects of bromfenac 0.1% and fluorometholone 0.1% on anterior chamber inflammation were equivalent; even on the basis of the disappearance of inflammation in the anterior chamber (inflammation grade: zero) there was no difference between the groups.
- Dry eye signs such as tear breakup time, Oxford staining, and symptoms in the NSAID and steroid groups also showed similar improvements after cataract surgery. Moreover, the NSAID group showed earlier recovery of conjunctival hyperemia than the steroid group.
